# Heidenhain Disease: Challenging Diagnosis of a Creutzfeldt-Jakob Variant

**DOI:** 10.7759/cureus.115516

**Published:** 2026-08-31

**Authors:** Rafael s Brambilla, Pedro N Fortunato, Rosana C Maffeis, Caroline M Tapajós Bolzani, Hilton M da Silva Júnior

**Affiliations:** 1 Medicine, Pontifícia Universidade Católica de Campinas, Campinas, BRA; 2 Neurology and Neurosurgery, Hospital Municipal Dr. Mário Gatti, Campinas, BRA

**Keywords:** creutzfeldt-jakob disease, heidenhain's disease, heidenhain's variant, neurodegeneration, prion disease, visual symptoms

## Abstract

Creutzfeldt-Jakob disease (CJD) is a rare disorder caused by prions, characterized by rapid deterioration of cognitive and neurological functions. The Heidenhain variant is an uncommon form in which visual symptoms are prominent at onset due to involvement of the occipital cortex, leading to a delayed diagnosis. We present the case of a 67-year-old woman who initially exhibited decreased visual acuity, followed by difficulties in reaching for and identifying objects, experiencing optic apraxia, psychomotor slowing, perseverative behavior, and impaired judgment. Brain magnetic resonance imaging (MRI) showed diffusion restriction and bilateral cortical T2/fluid-attenuated inversion recovery (FLAIR) hypersignal. Additionally, cerebrospinal fluid (CSF) analysis tested positive for the 14-3-3 protein. The patient experienced a rapid neurological decline and passed away four months after the onset of symptoms. This case report highlights the critical need to recognize the Heidenhain variant in patients presenting with isolated complex visual disturbances, especially when accompanied by early cognitive impairment.

## Introduction

Creutzfeldt-Jakob disease (CJD) is a rapidly progressive, prion-mediated neurodegenerative disorder classified among the transmissible spongiform encephalopathies [1]. Its pathogenesis is characterized by the conformational conversion of the cellular prion protein (PrPC), a glycoprotein constitutively expressed on neuronal cell surfaces, into the misfolded pathogenic isoform, PrPSc. This process promotes the accumulation of PrPSc within the central nervous system, resulting in neuronal loss, spongiform degeneration, and gliosis [2].

Its etiology is heterogeneous, encompassing sporadic, inherited, and acquired forms. Sporadic CJD (sCJD) is the predominant form, accounting for approximately 85% of all cases. Inherited CJD is associated with pathogenic mutations in the PRNP gene, located on the short arm of chromosome 20, which encodes the PrPC [3]. Acquired forms include variant CJD (vCJD), primarily associated with zoonotic transmission of the bovine spongiform encephalopathy agent to humans, and iatrogenic CJD (iCJD), resulting from exposure to prion-contaminated biological materials [3,4]. There is no evidence that CJD is transmitted through routine social or clinical contact between individuals.

The incidence of CJD is estimated to be one to two cases per million people per year [5]. It does not have a gender predilection but predominantly affects individuals between the fifth and eighth decades of life [3,5]. The clinical presentation is heterogeneous and may include myoclonus, neuropsychiatric manifestations, ataxia, visual disturbances, and akinetic mutism [3-5]. The diagnosis is supported by characteristic findings on electroencephalography (EEG) and magnetic resonance imaging (MRI), as well as cerebrospinal fluid (CSF) abnormalities, particularly elevated levels of 14-3-3 and tau proteins [5].

The Heidenhain variant represents a distinct clinical phenotype of sCJD, characterized by predominant involvement of the parieto-occipital lobes, particularly the primary visual cortex, leading to early visual symptoms that may precede the development of myoclonus, cognitive decline, and progressive dementia [1,2,4].

In the present report, we describe a rare case of Heidenhain variant Creutzfeldt-Jakob disease (HvCJD) in a 67-year-old woman with an unremarkable medical history, highlighting its clinical manifestations and diagnostic challenges, along with a brief review of the literature.

Part of this study was previously presented in abstract and poster form at the XII Congresso Paulista de Neurologia (May 29, 2019), the XXIX Congresso Brasileiro de Neurologia (September 6, 2021), the 22nd Samuel Pessoa Medical Academic Pre-Congress (October 8, 2024), the XXXI Congresso Brasileiro de Neurologia (October 19, 2024), the 13th Congresso Paulista de Clínica Médica (November 23, 2024), and the XV Congresso Paulista de Neurologia (June 7, 2025). This manuscript represents a substantially expanded and updated version of the work previously presented.

## Case presentation

A 67-year-old woman with an unremarkable medical history was admitted to the hospital with a reported one-month history of "dementia". A comprehensive history obtained from her family established that she had remained in her usual state of health until approximately three months before admission, when she developed decreased visual acuity. Over the ensuing months, she presented with progressive difficulty reaching for and identifying objects, psychomotor slowing, repetitive behaviors, and impaired judgment. Her son documented the clinical evolution in videos recorded on his mobile phone.

Neurological examination at admission revealed significant visual and cognitive impairment. The patient required assistance from family members to enter the examination room and was unable to identify the chair before sitting. She was alert but exhibited impaired attention, limited cooperation, and an inability to provide a reliable history. Repeated prompting was required to follow simple commands. Pupillary light reflexes and extraocular movements were preserved, and spontaneous antigravity movements were symmetric in all four extremities. Severe visual impairment was evident, with inability to recognize objects and optical apraxia. Despite the extent of the visual deficit, she appeared unaware of the impairment and attempted to ambulate without assistance.

Within the first hours of hospitalization, MRI and EEG were performed. The initial MRI was unremarkable, whereas EEG demonstrated diffuse slowing of the background activity. 

In view of the subacute clinical presentation, an extensive investigation for encephalitis was undertaken concurrently. CSF polymerase chain reaction (PCR) testing for herpes simplex virus types 1 and 2 (HSV-1/HSV-2), varicella-zoster virus (VZV), enterovirus, human herpesvirus 6 (HHV-6), cytomegalovirus (CMV), and Epstein-Barr virus (EBV), together with HIV-1/2 testing, syphilis serology, and arbovirus testing for dengue, Zika, and chikungunya, yielded no significant abnormalities. Empirical treatment with acyclovir and intravenous methylprednisolone pulse therapy was initiated; however, no clinical improvement was observed.

During the ensuing two weeks, the patient developed intermittent myoclonus and abulia. Given the clinical progression, additional diagnostic studies were pursued. Fifteen days after admission, a repeat brain MRI demonstrated restricted diffusion and cortical T2/FLAIR hyperintensity in both cerebral hemispheres (Figure 1). At the same time, EEG revealed generalized periodic discharges (Figure 2), and CSF analysis was positive for 14-3-3 protein.

**Figure 1:**
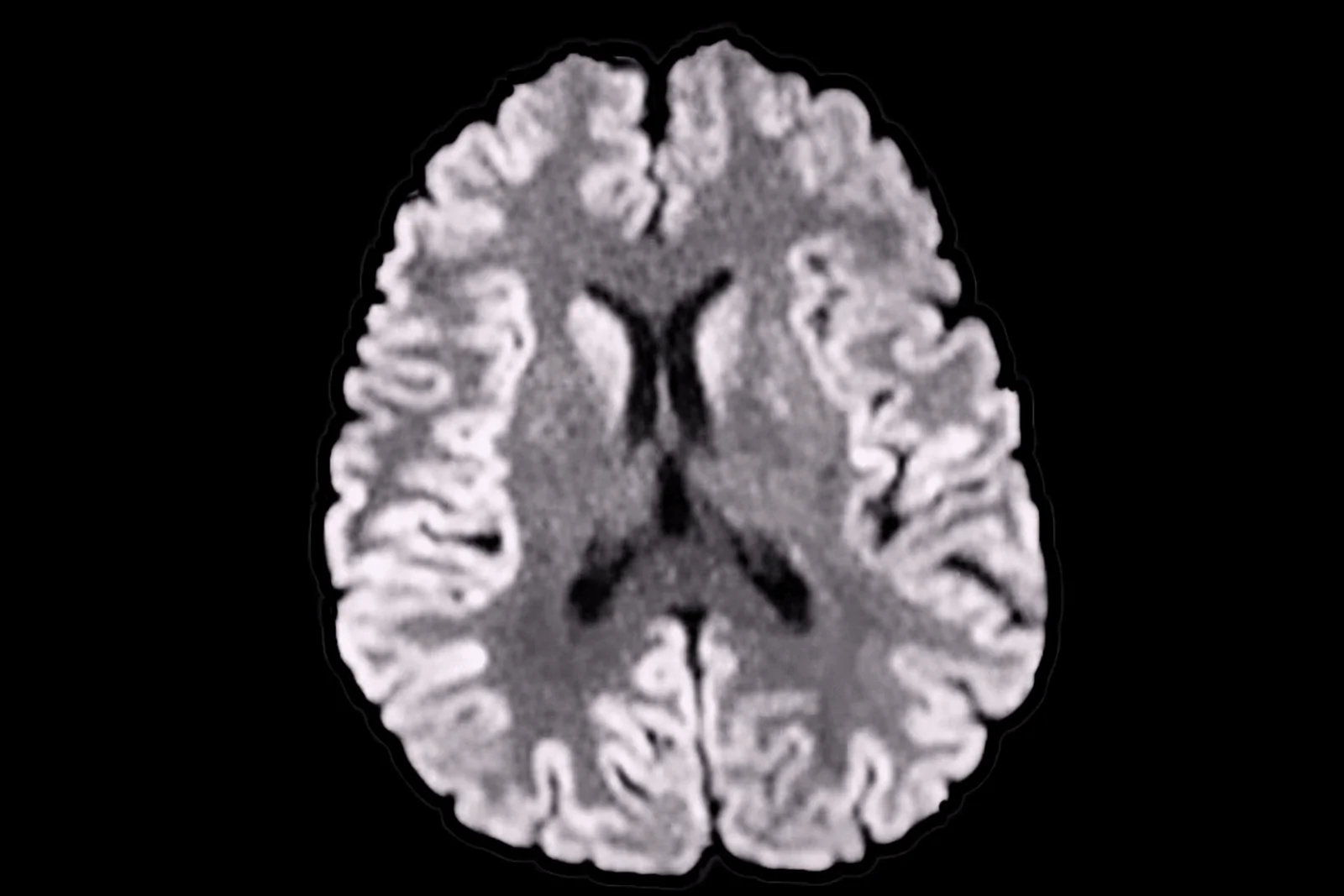
Repeat brain MRI demonstrating bilateral cortical abnormalities Brain MRI performed 15 days after admission, approximately three and a half months after symptom onset, demonstrating restricted diffusion and cortical T2/FLAIR hyperintensity involving both cerebral hemispheres.

**Figure 2:**
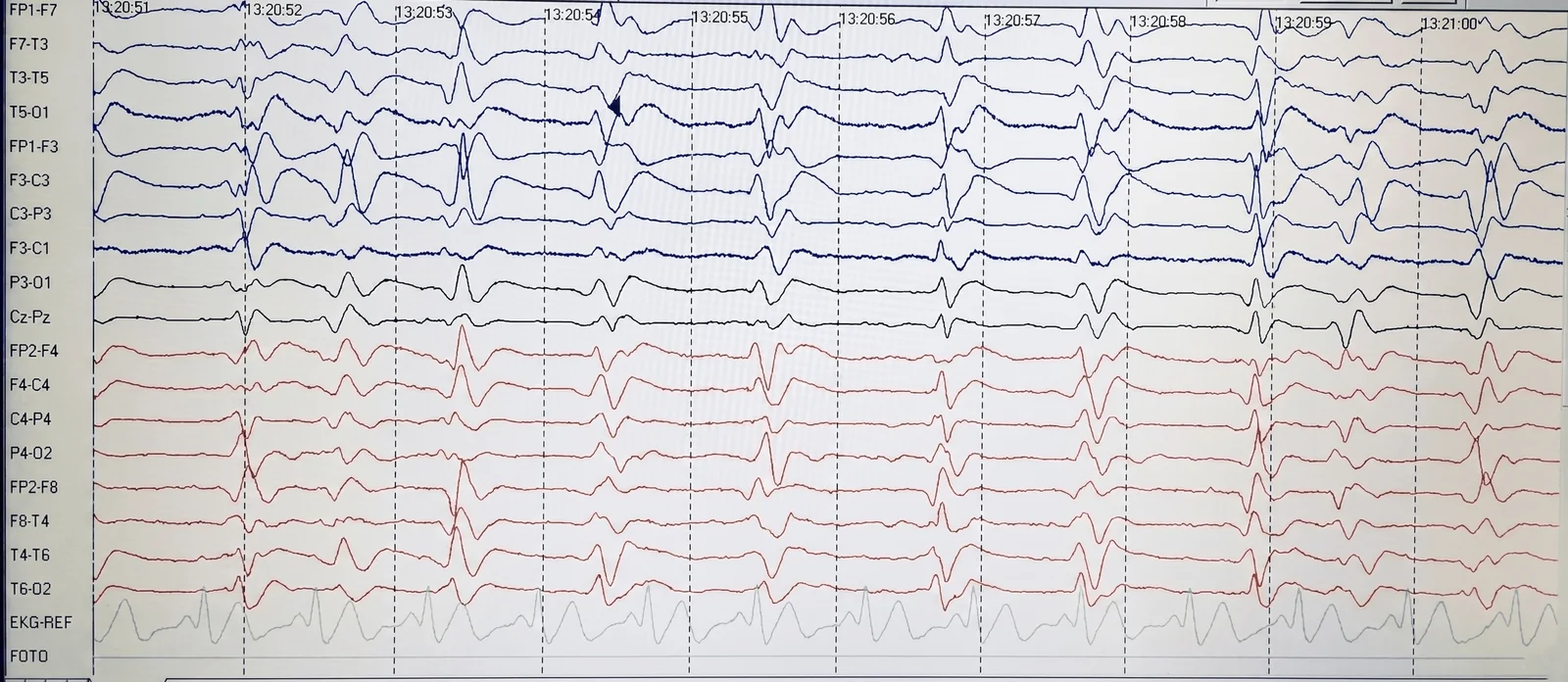
Repeat EEG demonstrating generalized periodic discharges EEG performed 15 days after admission, approximately three and a half months after symptom onset, demonstrating generalized periodic discharges.

Taken together, the rapidly progressive clinical course, initial visual manifestations, and characteristic MRI, EEG, and CSF findings supported the diagnosis of Heidenhain variant Creutzfeldt-Jakob disease.

Palliative care was subsequently instituted. The patient died four months after symptom onset due to complications of pneumonia. Written informed consent was obtained from her family for publication of the case and use of the recorded videos.

## Discussion

We report a case of HvCJD. This sCJD phenotype results from predominant parieto-occipital cortical involvement and is characterized by visual disturbances that precede psychomotor deterioration by variable periods [1-4]. Common manifestations include blurred vision, visual hallucinations, impaired identification of objects and colors, cortical blindness, and visual agnosia, followed by myoclonus, neuropsychiatric manifestations, ataxia, and akinetic mutism as the disease progresses [3,6]. Our patient initially presented with decreased visual acuity, followed by difficulty reaching for and identifying objects and optic apraxia, which rapidly progressed to psychomotor slowing, repetitive behaviors, and impaired judgment. Video recordings provided additional information on the clinical evolution and supported a cortical rather than exclusively ophthalmological origin of the visual impairment. 

HvCJD has been described predominantly in individuals in the fifth to eighth decades of life, consistent with our patient's age [5]. Visual symptoms frequently represent the initial manifestation, and affected individuals may therefore undergo ophthalmological evaluation before a neurological disorder is suspected. Early neuropsychomotor abnormalities may be subtle, leading clinicians to consider alternative diagnoses rather than CJD initially. As the disease progresses, the emergence of characteristic neuroimaging and electrophysiological abnormalities may raise suspicion for prion disease and prompt a more targeted diagnostic investigation [1-4,6]. This sequence was also observed in our patient and illustrates how the initial visual presentation may contribute to diagnostic delay. 

Regarding complementary examinations, HvCJD typically presents on brain MRI with cortical T2/FLAIR hyperintensity and diffusion restriction, with relative sparing of the subcortical white matter and basal ganglia [4]. Importantly, these abnormalities may not be detectable during the early stages of the disease [7,8]. A systematic review of 73 patients with HvCJD found that approximately 15% had no MRI abnormalities, emphasizing that an initially unremarkable examination does not exclude the diagnosis [8]. EEG may demonstrate periodic sharp-wave complexes or generalized periodic discharges, although these findings have limited sensitivity and specificity [9,10]. CSF biomarkers can provide additional diagnostic support. The 14-3-3 protein, which reflects rapid neuronal injury, has an estimated sensitivity of 92% and specificity of 80%, whereas total tau has reported sensitivities and specificities of 91.3% and 78.7%, respectively [11,12]. 

In our patient, the initial MRI was unremarkable; however, repeat imaging subsequently demonstrated characteristic cortical abnormalities. At the same time, EEG revealed generalized periodic discharges, and CSF analysis was positive for 14-3-3 protein. In the context of rapidly progressive neurological deterioration, these findings supported a probable diagnosis of sporadic CJD presenting as the Heidenhain variant, consistent with the 2017 International CJD Surveillance Network criteria [13]. 

Recent advances in prion amplification techniques, particularly protein misfolding cyclic amplification (PMCA) and real-time quaking-induced conversion (RT-QuIC), have improved the reliability of premortem diagnosis of prion diseases. For sCJD, RT-QuIC has demonstrated approximately 80-90% sensitivity and near 100% specificity [14,15]. However, its availability remains limited in some settings, restricting its broader clinical implementation. In the present case, the assay was not available at the treating institution, representing an additional limitation during the diagnostic evaluation. Neuropathological examination remains the definitive diagnostic standard, demonstrating neuronal loss, spongiform changes, astrocytosis, and PrPSc deposition [3].

The predominantly visual presentation of HvCJD may mimic several conditions associated with rapidly progressive cognitive or visual impairment, including autoimmune encephalitis, status epilepticus, hypoxic-ischemic encephalopathy, severe hypoglycemia, posterior cortical atrophy, hyperammonemia, and frontotemporal dementia [4]. Other causes of rapidly progressive dementia, such as cerebral vasculitis, cerebral amyloid angiopathy, and intravascular lymphoma, should also be considered [16]. In our patient, the initial diagnostic evaluation prioritized subacute infectious etiologies given the clinical presentation and epidemiological context. The absence of evidence for an infectious process and the lack of clinical response to empirical therapy progressively reduced the likelihood of these alternative diagnoses. 

Treatment of CJD remains predominantly supportive, as no disease-modifying therapy has demonstrated efficacy to date. Management should be individualized according to symptom burden, functional status, and the patient's goals of care. Multidisciplinary supportive measures may include physiotherapy, speech and language therapy, occupational therapy, nutritional assessment, and swallowing support. Pharmacological interventions are primarily directed toward symptom relief and may include antiseizure medications for myoclonus, antipsychotic agents for clinically significant behavioral disturbances, and analgesics for pain management [17]. 

Despite the eventual diagnosis, the patient continued to experience rapidly progressive neurodegeneration. Although survival in CJD is generally limited to approximately twelve months, our patient died four months after symptom onset, underscoring the aggressive course of HvCJD [3]. 

## Conclusions

As it is a potentially transmissible disease with an unfavorable prognosis, HvCJD should be rigorously considered in the differential diagnosis of patients presenting with isolated visual disturbances, particularly when such findings coexist with early cognitive impairment. Furthermore, new diagnostic methods may enhance the accuracy and timeliness of detection, thereby supporting appropriate clinical management. Continued research and heightened clinical awareness are essential to improve diagnostic precision and ultimately optimize patient outcomes.

## References

[1] Esezoobo O, Gosal D, Markey K (2026). Unexplained acute homonymous hemianopia as a presentation of Creutzfeldt-Jakob disease. Cureus.

[2] Madjer N, Shaju R, Vipond C, MacDougall A, Murty P (2024). Heidenhain variant of Creutzfeldt-Jakob disease: a case report. Cureus.

[3] Tokumitsu K, Oyama S, Takeuchi J, Yachimori K, Sugawara N, Yasui-Furukori N, Shimoda K (2021). Heidenhain variant of Creutzfeldt-Jakob disease resembles dementia with Lewy bodies. Psychogeriatrics.

[4] Muniz BC, Makita LS, Ribeiro BN, Marchiori E (2019). The Heidenhain variant of Creutzfeldt-Jakob disease. Radiol Bras.

[5] Brasil. Ministério da Saúde. Secretaria de Vigilância em Saúde (2018). Notification and investigation protocol for Creutzfeldt-Jakob disease with a focus on identifying the new variant. Brasília-DF: Ministério da Saúde.

[6] Obergassel J, Lohmann L, Meuth SG, Wiendl H, Grauer O, Nelke C (2020). An enigmatic case of cortical anopsia: antemortem diagnosis of a 14-3-3 negative Heidenhain-variant MM1-sCJD. Prion.

[7] Ponnam HC, Sonavane K, Shirsat P, Gopagoni R, Sakamuri L, Kashyap R (2025). Creutzfeldt-Jakob disease: insights from a case report and diagnostic challenges. Cureus.

[8] Muthusamy S, Garg P, Chandra RV, Seneviratne U (2021). How common are seizures in the Heidenhain variant of Creutzfeldt-Jakob disease? A case report and systematic review. J Clin Neurosci.

[9] Macfarlane RG, Wroe SJ, Collinge J, Yousry TA, Jäger HR (2007). Neuroimaging findings in human prion disease. J Neurol Neurosurg Psychiatry.

[10] Smith SJ (2005). EEG in neurological conditions other than epilepsy: when does it help, what does it add?. J Neurol Neurosurg Psychiatry.

[11] Muayqil T, Gronseth G, Camicioli R (2012). Evidence-based guideline: diagnostic accuracy of CSF 14-3-3 protein in sporadic Creutzfeldt-Jakob disease: report of the guideline development subcommittee of the American Academy of Neurology. Neurology.

[12] Yang SL, Zhang LY, Zhang SF (2022). Clinical and prognostic features of Heidenhain variant of Creutzfeldt-Jakob disease: a retrospective case series study. Eur J Neurol.

[13] Watson N, Hermann P, Ladogana A (2022). Validation of revised International Creutzfeldt-Jakob disease surveillance network diagnostic criteria for sporadic Creutzfeldt-Jakob disease. JAMA Netw Open.

[14] Jurcau MC, Jurcau A, Diaconu RG, Hogea VO, Nunkoo VS (2024). A systematic review of sporadic Creutzfeldt-Jakob disease: pathogenesis, diagnosis, and therapeutic attempts. Neurol Int.

[15] Baban J, Saeed A, Thura K, Thida Nwe H, Diaconu C (2025). Atypical presentation of Creutzfeldt-Jacob disease (CJD): early incontinence and delayed imaging in a rapidly progressive dementia. Cureus.

[16] Geschwind MD (2016). Rapidly progressive dementia. Continuum (Minneap Minn).

[17] Ng M, Grundy A, Appleby B (2026). Multidisciplinary recommendations for palliative and supportive care in Creutzfeldt-Jakob disease and related disorders. Age Ageing.

